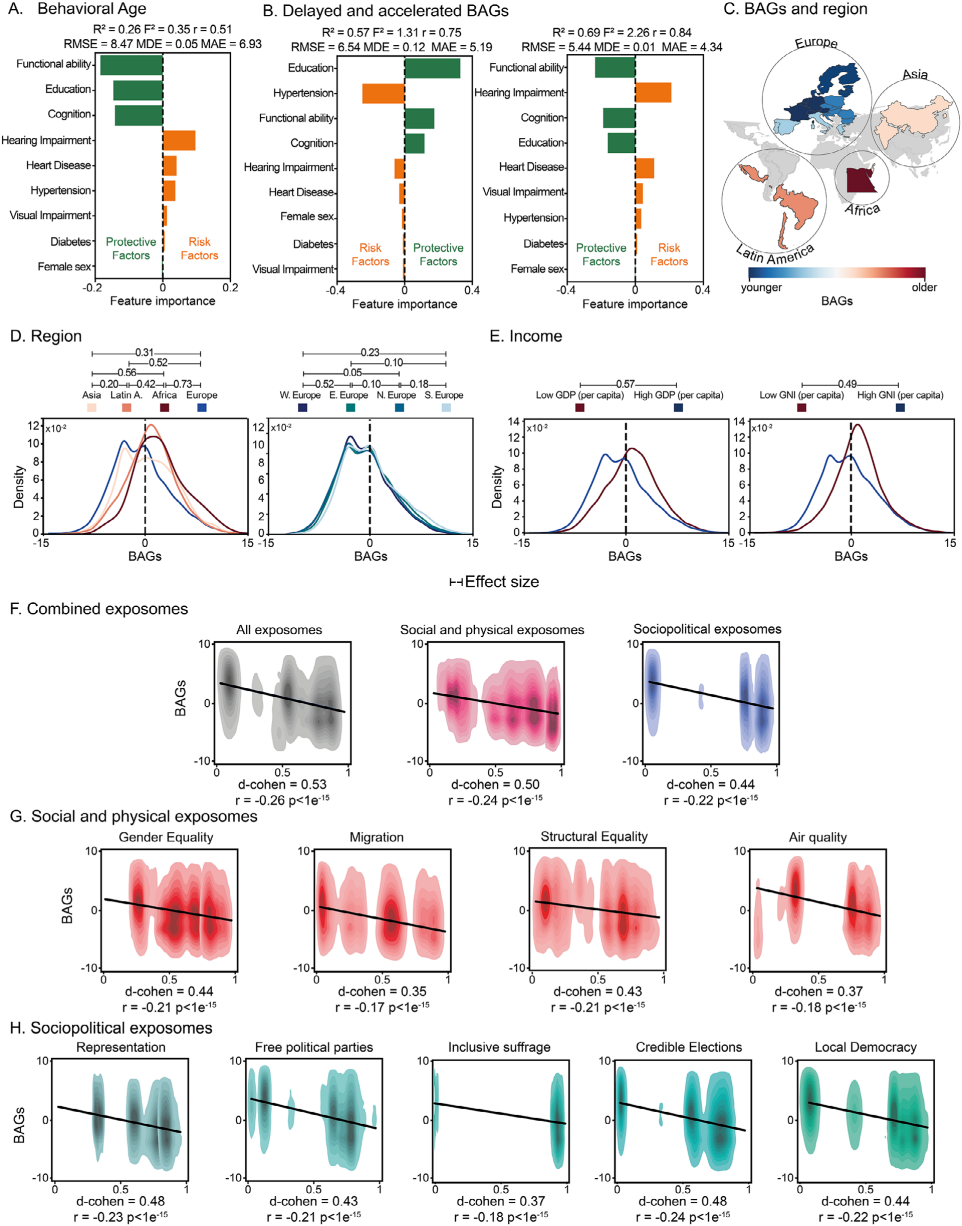# Predicting accelerated and delayed aging in global settings: biobehavioral age gaps and the role of global exposomes

**DOI:** 10.1002/alz70860_099534

**Published:** 2025-12-23

**Authors:** Hernan Hernandez, Hernando Santamaria‐Garcia, Sebastian Moguilner, Agustina Legaz, Pavel Prado, Jhosmary Cuadros, Liset Gonzalez, Raul Gonzalez‐Gomez, Joaquín Migeot, Carlos Coronel‐Oliveros, Enzo Tagliazucchi, Marcelo Adrian Maito, Maria Eugenia Godoy, Josephine Cruzat, Ahmed Shaheen, Temitope Hannah Farombi, Daniel Salazar, Lucas Uglione Da Ros, Wyllians Vendramini Borelli, Eduardo R. Zimmer, Alfred Kongnyu NJAMNSHI, Swati Bajpai, A.B Dey, Cyprian M Mostert, Zul Merali, Mohamed Salama, Sara Ayman Moustafa, Francesca R Farina, Sol Fittipaldi, Florencia Altschuler, Vicente Medel, David Huepe, Kristine Yaffe, Chinedu T Udeh‐Momoh, Harris A Eyre, Pawel Swieboda, Brian Lawlor, Jaime Miranda, Claudia Duran‐Aniotz, Sandra Baez, Agustin Ibanez

**Affiliations:** ^1^ Latin American Brain Health Institute (BrainLat), Universidad Adolfo Ibañez, Santiago, Chile; ^2^ Pontificia Universidad Javeriana, Bogotá, Colombia; ^3^ Global Brain Health Institute (GBHI), University of California San Francisco (UCSF); & Trinity College Dublin, San Francisco, CA, USA; ^4^ Latin American Brain Health Institute (BrainLat), Universidad Adolfo Ibáñez, Santiago, Región Metropolitana de Santiago, Chile; ^5^ Harvard Medical School, Boston, MA, USA; ^6^ Cognitive Neuroscience Center (CNC), Universidad de San Andrés, Buenos Aires, Buenos Aires, Argentina; ^7^ Escuela de Fonoaudiología, Facultad de Odontología y Ciencias de la Rehabilitación, Universidad San Sebastián, Santiago, Chile; ^8^ Facultad de Ingeniería, Universidad de Concepción, Concepción, Chile, Concepcion, Chile; ^9^ Centro Interdisciplinario de Neurociencia de Valparaíso (CINV), Valparaíso, Chile; ^10^ Latin American Brain Health Institute (BrainLat), Universidad Adolfo Ibañez, Santiago de Chile, Chile, Santiago, Chile; ^11^ Alexandria Faculty of Medicine, Alexandria, Egypt, Alexandria, Egypt; ^12^ University College Hospital, Ibadan, Nigeria; ^13^ London School of Economics and Political Science, London, United Kingdom; ^14^ Universidade Federal Do Rio Grade Do Sul, Porto Alegre, Rio Grande do Sul, Brazil; ^15^ Brain Institute of Rio Grande do Sul (InsCer), PUCRS, Porto Alegre, Rio Grande do Sul, Brazil; ^16^ Universidade Federal do Rio Grande do Sul, Porto Alegre, Rio Grande do Sul, Brazil; ^17^ Brain Research Africa Initiative, Yaounde, Centre, Cameroon; ^18^ Department of Geriatric Medicine, All India Institute of Medical Sciences, New Delhi, India; ^19^ Department of Geriatrics, All India Institute of Medical Sciences, New Delhi, India; ^20^ Aga Khan University, The Brain and Mind Institute, Nairobi, Kenya; ^21^ Brain and Mind Institute, Aga Khan University, Nairobi, Kenya; ^22^ Institute of Global Health and Human Ecology at the American University in Cairo, Cairo, Cairo, Egypt; ^23^ The American University in Cairo, Cairo, Egypt; ^24^ Global Brain Health Institute, Trinity College Dublin, Dublin, Ireland; ^25^ Universidad Adolfo Ibáñez, Santiago de Chile, Chile; ^26^ University of California San Francisco / San Francisco VA Medical Center, San Francisco, CA, USA; ^27^ Aga Khan University Brain and Mind Institute, Nairobi, Nairobi, Kenya; ^28^ Neuro‐Policy Program, Center for Health and Biosciences, Baker Institute for Public Policy, Rice University, Houston, Texas, Texas, United States Minor Outlying Islands; ^29^ International Center for Future Generations (ICFG), Brussels, Belgium, Brussels, Belgium; ^30^ Sydney School of Public Health, Faculty of Medicine and Health, University of Sydney, Camperdown, New South Wales, Australia, Sydney, Australia; ^31^ Universidad de los Andes, Bogotá, Colombia, Bogota, Bogota, Colombia; ^32^ Global Brain Health Institute (GBHI), Trinity College Dublin, Dublin, Dublin, Ireland; ^33^ Global Brain Health Institute (GBHI), University of California San Francisco (UCSF); & Trinity College Dublin, Dublin, Ireland

## Abstract

**Background:**

Global health challenges like aging and dementia are shaped by socioeconomic disparities, environmental factors, and social determinants of health. We developed a behavioral age gap (BAG), measuring the difference between expected behavioral age and chronological age

**Method:**

We utilized a cross‐sectional sample (*n* = 161,981) comprising countries from Latin America, Europe, Asia, and Africa. Behavioral age was estimated using a Gradient Boosting Regressor with 10‐fold cross‐validation, incorporating multiple risk factors (hypertension, diabetes, heart disease, female sex, visual impairment and hearing impairment) and protective factors (cognition, functional ability, education) associated with healthy aging. BAG was calculated as the difference between predicted and chronological age, and adjusted gaps were derived from the residuals of regressing BAG on chronological age.

**Result:**

Chronological age was accurately estimated using biobehavioral predictors. Key protective predictors were functional ability, education, and cognition, while main risks were hearing impairment, heart disease, and hypertension. Participants were categorized into delayed or accelerated aging groups to explore biobehavioral factors in aging. Both models demonstrated high predictive accuracy, especially for accelerated aging. BAGs varied significantly across regions and income levels, increasing from Europe to Asia, LA, and Africa. Participants from LIC displayed accelerated aging compared to HIC. Adverse exposomes were linked to accelerated aging with large effect sizes.

**Conclusion:**

This work positions BAGs as markers of aging disparities, emphasizing the influence of inequalities and exposomes, while providing measures for targeted interventions and research.